# Indocyanine Green Angiography in the Evaluation of Surgical Amputation Level in Patients with Arterial Ulcers Due to Thromboangiitis Obliterans

**DOI:** 10.3390/medicina61091678

**Published:** 2025-09-16

**Authors:** Simay Akyüz, Hikmet Erhan Güven, Kerim Bora Yılmaz

**Affiliations:** 1 Department of Oncology Nursing, Gulhane Faculty of Nursing, University of Health Sciences, Ankara 06010, Turkey; 2 Department of General Surgery, Diabetic Foot and Chronic Wounds, University of Health Sciences, Etlik City Hospital, Ankara 06010, Turkey; 3 Department of General Surgery, University of Health Sciences Gulhane Training and Research Hospital, Ankara 06010, Turkey

**Keywords:** indocyanine green angiography, arterial ulcers, thromboangiitis obliterans

## Abstract

*Background and Objectives*: The aim of this study was to evaluate the role of Indocyanine Green Angiography (ICGA) on amputation levels and the wound healing process in patients with thromboangiitis obliterans (TAO) and complex diabetes. *Materials and Methods*: The research was conducted on 26 inpatients, with TAO and lower extremity ulcers, who were treated between November 2019 and September 2021. A retrospective analysis was made of overall health status, wound characteristics, and surgical outcomes. *Results*: The patients comprised 88.5% males and 11.5% females, with a mean age of 62.31 years. In 84.6% of the patients evaluated with ICG angiography, the wound healing process was achieved with no complications. Negative and positive correlations were identified between ICG density values and wound healing time, disease-free follow-up time, and ICG full density time. *Conclusions*: These findings suggest that ICG angiography may assist in achieving safe surgical margins for patients with TAO. In conclusion, ICG angiography can be considered a valuable predictive tool for assessing tissue perfusion in TAO-related lower extremity ulcers.

## 1. Introduction

Lower extremity ulcers are common conditions that can lead to amputation, exhibit delayed healing, and negatively impact quality of life [1]. The ulcer etiology often includes various risk factors, such as diabetes-related vasculopathy, atherosclerosis, peripheral neuropathy, and non-atherosclerotic inflammatory arteritis [2]. Clinical manifestations of lower extremity vasculopathy often precede the development of ulcers, resulting in a lack of appropriate diagnosis and treatment for such vascular conditions [1]. This underscores the importance of accurately diagnosing the etiology of ulcers. In long-term tobacco users, arterial ulcers related to thromboangiitis obliterans may resemble complicated diabetic foot ulcers, which can influence clinical and surgical approaches.

Thromboangiitis obliterans is a non-atherosclerotic, thrombotic, segmental inflammatory disease affecting small and medium-sized arteries in the lower extremities [3,4]. Tobacco use is the primary etiology for the onset and progression of this rare disease [5,6]. Current diagnosis of TAO uses the Shionoya and Olin criteria [3,7], which include parameters such as onset before age 50, a history of tobacco use, ischemia of the lower extremities, normal proximal artery segments, and the absence of vasculitis findings. Differential diagnoses should evaluate atherosclerotic peripheral arterial disease, embolic events, vasculitis, and hypercoagulability [8]. Meeting the majority of the clinical and angiographic diagnostic criteria is sufficient for a definitive diagnosis [9].

Korea, India, and Japan have the highest incidence of TAO, while in Western Europe, rates range from 0.5% to 5.6% [3]. Although previous literature has suggested that TAO is predominantly seen in young adult males, it has recently been observed that tobacco use among women has led to a significant increase in TAO rates [10]. Patients often present with symptoms of lower or upper extremity ischemia, including intermittent claudication, superficial thrombophlebitis, limb coldness, pain at rest, ulcers, and gangrene [11].

Traditional diagnostic tools for TAO include non-invasive diagnostic tests such as the ankle-brachial index (ABI), toe pressure and toe-brachial index (TBI), transcutaneous oxygen pressure (TcPO2), and continuous wave Doppler ultrasound (CWD) [12]. ABI is known for reliability stemming from conditions such as medial calcinosis [13,14]. TcPO2, valued for its simplicity and non-invasiveness, serves as a commonly employed diagnostic tool for evaluating perfusion status and wound healing potential in diabetic patients. However, it has notable disadvantages, including sensitivity to factors such as skin condition, edema, infection, and movement, leading to variability in results [15,16]. Furthermore, TcPO2 provides only localized assessments and does not directly evaluate blood flow [8]. When these non-invasive methods are insufficient or inconclusive, imaging modalities such as Duplex Ultrasound (DUS), Computed Tomography Angiography (CTA), Magnetic Resonance Angiography. The lack of sufficient accuracy of DUS creates the need for additional imaging techniques [12]. CTA has good accuracy, but may be a relative contraindication in cases of allergy because of the use of ionizing radiation and iodine contrast [17]. MRA is an imaging technique with high sensitivity and specificity. However, it requires long image acquisition times, is not available in all centers, and cannot be used in noncompliant patients [18]. While these advanced imaging modalities provide detailed anatomical assessment, they primarily offer morphological information and may not adequately reflect tissue-level microvascular perfusion, which is particularly relevant in TAO where skip lesions and collateral circulation patterns are characteristic features.

Amputations related to TAO occur at a frequency 70% higher than those due to atherosclerotic ischemia, as the disease primarily affects distal arteries, making reconstructive surgeries rare with typically low success rates [19]. While initially symptomatic in a single extremity, TAO can progress proximally and affect multiple extremities [3]. The preservation of functional limbs is critical to maintaining quality of life for patients [20].

The high revision rates and prolonged healing times associated with traditional amputations have adverse effects on rehabilitation. Nevertheless, surgical interventions such as revascularization and sympathectomy have demonstrated potential to enhance peripheral blood flow and reduce amputation rates. Early diagnosis and prompt intervention can alleviate debilitating symptoms and reduce the risk of major amputations [8].

Vascular assessment is crucial in predicting the likelihood of wound healing and amputation risk in patients with TAO-related lower extremity ulcers. Recently, innovative methods such as ICG fluorescence angiography have been used to evaluate tissue perfusion in various medical fields, offering rapid, non-invasive, and reproducible assessment of perfusion following intravenous ICG dye injection, which fluoresces in the near-infrared spectrum [21]. Due to its low side effect profile and the absence of nephropathy risk, particularly advantageous for diabetic patients, ICG fluorescence angiography can also be used to assess tissue perfusion, minimize dehiscence, delineate safe surgical margins, predict wound healing, and reduce rates of readmission and revision surgery in patients with TAO. Studies specifically addressing the use of ICG in patients with TAO are scarce in the literature, and objective data regarding its application in limb salvage procedures are limited [19]. The aim of this study is to investigate the role of ICG angiography in amputation rates and wound healing in patients with TAO and complex diabetes. Additionally, this study seeks to lay the groundwork for more comprehensive future research on the role of ICG angiography in the management of TAO and to provide preliminary data on its potential benefits.

## 2. Method

This retrospective analysis was conducted in the Diabetic Foot Clinic of a Training and Research Hospital. The study sample comprised 26 inpatients diagnosed with diabetes and TAO, treated for foot ulcers between November 2019 and September 2021.

**Figure 1:**
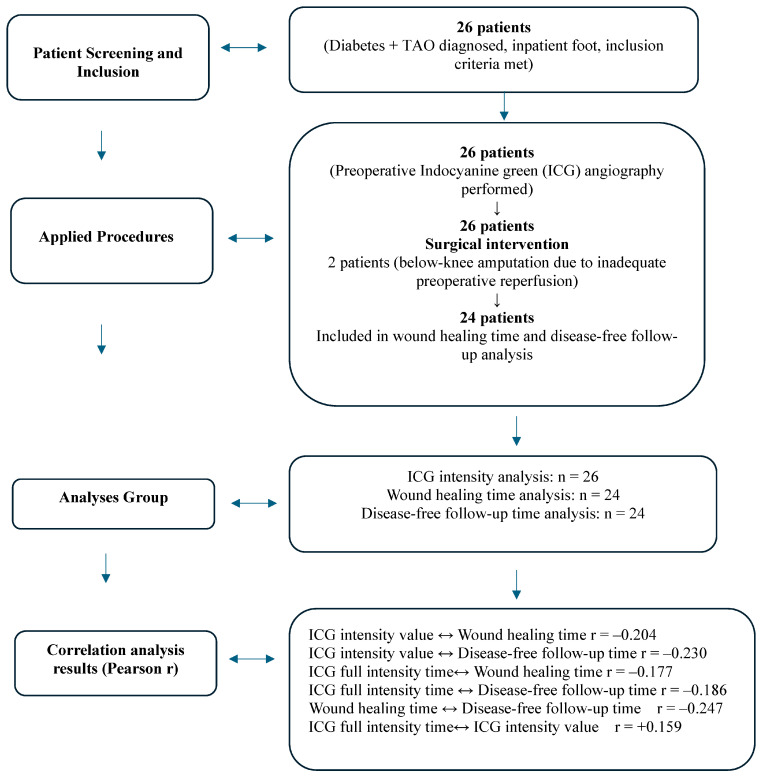
Number of patients included in the study and analysis groups.

The study inclusion criteria were defined as:

(a) Admission due to foot ulcers, (b) History of tobacco use (smoking or smokeless tobacco), (c) Clinical findings consistent with TAO including: Distal extremity ischemia (rest pain, claudication, cold skin), Digital ischemia or ulceration in segmental distribution, Cold extremities with diminished or absent pulses (d) Diagnosis of TAO confirmed by comprehensive evaluation including: -Arterial Doppler ultrasonography showing characteristic segmental arterial occlusions in small and medium-sized vessels with collateral circulation patterns, -Laboratory exclusion of vasculitic and hypercoagulable conditions, (e) Exclusion of other causes of arterial insufficiency (atherosclerotic disease, autoimmune vasculitis, hypercoagulable states), (f) Absence of allergies to sodium iodide, iodide, or ICG, (g) Concurrent diabetes mellitus was not considered an exclusion criterion when other TAO diagnostic features were present, or ICG The number of patients included in the study and the analysis groups are presented in the Figure 1.

### 2.1. Ethical Considerations

Approval for the study was received from the Ethics Committee on 27 February 2024, under decision number 2024-70.

### 2.2. Research Implementation Process

Following ethical approval, demographic data, wound classifications on admission, peripheral endovascular angiography results, ICG angiography data, surgical types, complications, repeat surgeries, and healing times were systematically recorded and analyzed.

### 2.3. ICG Fluorescence Imaging

Indocyanine Green Angiography (ICG) is a contemporary technique that allows for the real-time intraoperative assessment of local vascularization. ICG is a fluorescent, water-soluble, non-radioactive, and non-toxic green contrast agent. Upon intravenous injection, it rapidly binds to plasma proteins, is swiftly cleared by the liver, and excreted via bile. These properties validate its use in angiographic evaluations.

In this study, the images were acquired using the SPY Elite System^®^ (Stryker, Kalamazoo, MI, USA). The SPY Elite System^®^ facilitates real-time evaluation of tissue microvascular perfusion through high-speed imaging following the intravenous administration of indocyanine green. The software associated with the SPY Elite^®^ System enables microvascular perfusion assessments utilizing perfusion contour maps immediately after the ICG injection [22].

Prior to imaging, the positions of the patient and the device are adjusted according to the target area. Initially, an ampule of IV antihistamine (Avil amp^®^, 45.5 mg/2 mL, Sandoz Pharmaceutical Industry and Trade Inc., İstanbul, Turkey) is administered. Subsequently, the patient receives 25 mg of ICG followed by a 10 mL intravenous saline flush.

Following the ICG administration, perfusion contour maps and fluorescence imaging are employed to record the intensities in the wound bed and surrounding tissues. A tissue area deemed to be 100% perfused is designated as the reference point based on the perfusion contour maps generated by the SPY Elite^®^ System software, V1.9.4.4680. This reference point is used to determine the relative perfusion percentages of adjacent regions. Tissue areas identified as having perfusion levels less than 20% on the perfusion contour map are classified as non-viable.

### 2.4. Statistical Analysis

ICG fluorescence intensity videos captured in the wound and surrounding areas were evaluated by an expert clinician. Areas exhibiting reduced fluorescence were marked as “low fluorescence,” while all other regions were labeled “normal fluorescence”. ICG saturation times and inflammation intensities with 20% sensitivity were recorded. Data analysis was performed using SPSS for Windows Version 25.0 software (IBM Corporation, Armonk, NY, USA). Conformity of data the data to normal distribution was assessed using the Shapiro–Wilk test, with distributions described as counts and percentages for discrete data and mean ± standard deviation values for continuous data. Pearson correlation analysis was applied to determine relationships between continuous variables, with an alpha level set at 0.05 for all analyses.

## 3. Results

In this study, the demographic and clinical parameters of 26 patients who presented with diabetic foot complaints and were diagnosed with TAO were comprehensively analyzed. The study cohort was predominantly male (88.5%) with a mean age of 62.31 ± 9.97 years. All the patients had a history of smoking and Type 2 diabetes.

Wound characteristics at presentation were classified according to the international diabetic foot classification, with 96.2% categorized as Wagner type 4 and 88.5% as PEDIS stage 3. According to the arterial Doppler USG results performed during clinical admission, peripheral angiography was performed in 88.5% (23) of the patients, and the rate of patients with patency reported in one or more arteries from the SFA in the proximal to the distal is 61.5% (16). The surgical interventions included forefoot amputation (38.5%) and minor amputations (34.6%). Of the patients evaluated with ICG angiography, 84.6% experienced an uncomplicated recovery process. The demographic and clinical characteristics of this study population are presented in Table 1.

**Figure 2:**
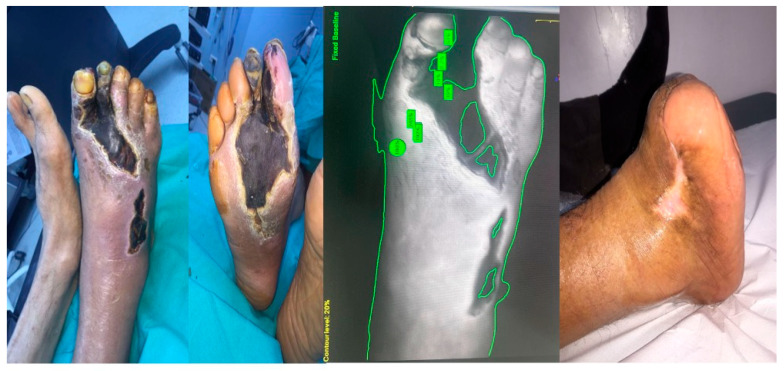
Example of ICGA evaluation and long-term follow-up results in the operating room.

The average ICG intensity value was recorded as 165.58 ± 27.6 (range, 123–236). The mean time for ICG to achieve maximum intensity in the wound bed and surrounding tissues was 27.46 ± 6.4 s (range, 17–45 s). Of the patients evaluated, two exhibited inadequate perfusion on preoperative ICG angiography and subsequently required below-knee amputation due to compromised postoperative healing and necrosis. In 24 patients, the mean duration for wound epithelialization was documented as 147.12 ± 107.36 days (range, 30–365 days) (Figure 2).

Long-term follow-up revealed the mean duration of disease-free follow-up to be 469.17 ± 206.48 days (range, 30–835 days), with no occurrences of new wound dehiscence or level elevation. The ICG angiography data evaluation results are summarized in Table 2.

The analyses revealed a negative correlation between ICG intensity values and wound healing times, disease-free follow-up duration, and ICG full intensity times (Table 3). A weak inverse correlation was determined between wound healing duration and ICG full intensity time with a value of −0.177, suggesting that prolonged ICG full intensity time may be correlated with a shorter wound healing time. The correlation between disease-free follow-up and ICG full intensity time was −0.186, also suggesting a weak negative relationship. The correlation value between disease-free follow-up and time to wound healing duration was −0.247, indicating that longer healing processes were correlated with shorter disease-free follow-up periods. The correlation between ICG full intensity time and ICG intensity value was 0.159, suggesting that an increase in ICG intensity could correspond with prolonged saturation. A weak negative correlation of −0.204 was established between wound healing time and ICG intensity value, highlighting that higher ICG intensity values could be correlated with extended healing times. The correlation value between disease-free follow-up and ICG intensity was determined to be −0.23, indicating a potential reduction in disease-free follow-up times in patients with elevated ICG density values. The correlation analysis results are presented in Table 3.

The findings illustrated weak predominantly negative correlations among the examined variables.

## 4. Discussion

The results of this preliminary study involving TAO patients evaluated with Indocyanine Green (ICG) angiography showed that 84.6% achieved wound healing without complications following surgery. Weak negative correlations were determined between wound healing time and ICG full intensity time, as well as between disease-free follow-up and ICG full intensity time, while weak positive correlations were seen between ICG intensity value and ICG full intensity time.

Previous reports have suggested that typical TAO clinical criteria include age under 45 years [3] or between 25 and 45 years. However, a recent retrospective analysis in Korea identified an average TAO patient age of 62.0 ± 15.7 years, with approximately 80% aged over 50 years [23].

The age of the current study participants was above average and all had a history of diabetes and tobacco use. These differences in patient characteristics could possibly be due to ethnic differences, patient health literacy, or limited access to healthcare.

Thromboangiitis obliterans is known to have a significant association with smoking [24]. Smoking contributes to the oxidative stress state in TAO, leading to cellular damage, thrombosis, and limb ischemia due to low antioxidant levels in smokers [25]. Given that all the current study patients were smokers, cessation of smoking from the time of diagnosis is imperative for preventing disease advancement and minimizing major amputation risks [24]. Healthcare professionals should engage patients in smoking cessation programs and establish effective education initiatives, potentially reducing TAO-related mortality and morbidity.

All patients had comorbid diabetes and wound classifications were determined according to diabetic foot wound standards. Diabetes is a chronic disease that exerts atherosclerotic effects on the vascular system, and comorbidities such as hypertension, myocardial infarction, and chronic kidney disease consequently increase the risks of TAO [23]. Predominantly, the ulcers were classified as Wagner type 4 and PEDIS stage 3, suggesting that the coexistence of these chronic conditions can precipitate significant tissue damage due to factors such as smoking history and poor glycemic control.

A large proportion of the current study patients underwent peripheral angiography, with over half successfully achieving partial or complete outcomes. A study conducted in 2017, which included 20 patients with TAO chronic limb ischemia and claudication, demonstrated a remarkable 96% success rate in 25 limbs following endovascular interventions [26]. Another similar investigation reported a technical success rate of 87.5% in 28 patients with TAO receiving endovascular recanalization [27]. The current study findings are consistent with the literature, underscoring the potential of diagnostic and therapeutic PTA in enhancing wound healing and establishing feasible surgical margins for patients with TAO. Suitable peripheral interventions may further mitigate surgical complications, safeguarding against recurrent surgeries and amputations.

Studies have confirmed that ICG angiography has a significant predictive value for postoperative wound complications in the lower extremities [28]. For example, a study examining the surgical stumps of 10 patients displaying ischemic tissue damage via ICG fluorescence three days post-surgery revealed that 60% exhibited primary healing potential, while 30% had indications for secondary amputations [29]. Our research results show that preoperative ICG angiography may contribute to favorable surgical outcomes and lower complication rates while positively affecting disease-free follow-up periods. Notably, 84.6% of the participants achieved wound healing despite some experiencing delayed healing and longer epithelialization times.

The analyses of this study suggest a duality of positive and negative correlations among ICG full intensity time, time to wound healing, disease-free follow-up duration, and ICG intensity value. These observations point to the potential for ICG angiography results to reflect disease progression and postoperative recovery, suggesting that timely perfusion may plausibly promote recovery in TAO patients, particularly considering the trend toward shorter wound healing times and longer times to full ICG intensity.

Based on the findings of this preliminary study, the observed negative correlation between wound healing time and disease-free follow-up suggests a potential association between prolonged healing processes and an increased risk of complications and recurrence in TAO patients and warrants further investigation and ongoing patient follow-up. The progressive nature of TAO can lead to episodic flares that can interrupt or complicate the healing process, necessitating adaptive treatment strategies and intensified surveillance protocols. While these preliminary observations highlight the importance of realistic patient counseling regarding expected healing timelines and the need for comprehensive follow-up programs to detect early signs of healing complications or disease recurrence, larger prospective studies are needed to confirm these findings and determine the clinical utility of ICGA in the management of TAO.

Additionally, a positive correlation between ICG intensity values and times at full ICG intensity may be related to disease severity. Higher intensities may indicate compromised or impaired healing processes. This, in line with the previous finding, suggests the importance of patient monitoring and vascular assessment.

Based on these preliminary findings, ICG angiography may provide useful information that can contribute to clinical management discussions in TAO patients, including surgical amputation levels. These observations suggest that disease-free follow-up periods may be associated with ICGA findings and wound care outcomes, potentially helping to protect patients with TAO-related ulcers from repeat amputations and maintaining surgical amputation margins within safer ranges. They also point to potential benefit in assessing non-viable wound bed tissue. However, these preliminary findings warrant further research through larger, controlled studies to determine whether ICGA can be validated as a reliable diagnostic tool in the management of TAO patients.

## 5. Conclusions

This preliminary study suggests that ICG angiography may have potential as a tool for preoperative tissue perfusion assessment in lower extremity ulcers associated with TAO.

While these preliminary findings suggest potential benefit in the real-time functional assessment of tissue perfusion and vascular systems in TAO patients, further research is needed to determine whether ICGA can reliably guide clinical decisions regarding amputation levels and wound care management. Future studies should investigate the clinical utility and standardization of ICGA protocols before they are implemented routinely in TAO patient care.

## 6. Limitations

While our research results are promising for a rare disease like TAO, further studies with larger cohorts are needed to ensure the statistical power and generalizability of the findings to larger populations. The retrospective and single-center nature of the study may present methodological limitations, particularly regarding selection and information biases, necessitating cautious interpretation of the results. Furthermore, our study was unable to control for confounding factors such as diabetes severity, glycemic control, nutritional status, and comorbidities, which may have influenced the correlations and results obtained. Therefore, prospective studies with larger sample sizes and control groups will fully demonstrate the potential of ICG angiography in TAO surgery and provide the necessary level of evidence for this technology.

## Figures and Tables

**Table 1:** The Demographic and Clinical Characteristics of the Patients with Lower Extremity Ulcers Related to Complex Diabetes and Thromboangiitis Obliterans.

Variables	Mean ± SD (Min–Max)	n (%)
**Age (years)**	62.31 ± 9.97	
**Gender**		
Female		3 (11.5)
Male		23 (88.5)
**Smoking history**		
Yes		26 (100.0)
**Presence of Diabetes**		
Yes		26 (100.0)
**Wound classification—WAGNER**		
Wagner Type 3		1 (3.8)
Wagner Type 4		25 (96.2)
**Wound classification—PEDIS**		
PEDIS Type 2		3 (11.5)
PEDIS Type 3		23 (88.5)
**Peripheral Endovascular Angiography**		
Performed		23 (88.5)
Not performed		3 (11.5)
**Success status of angiography**		
Successful		16 (61.5)
Not successful		7 (26.9)
**Surgery performed**		
Debridement		1 (3.8)
Minor Amputation		9 (34.6)
Minor Amputation and Debridement		6 (23.1)
Forefoot amputation		10 (38.5)
**Postoperative Flap Necrosis**		
Yes		4 (15.4)
No		22 (84.6)
**Wound Complications**		
None		15 (57.7)
Delayed wound healing		7 (26.9)
Flap necrosis		4 (15.4)
**Repeated surgery/Re-amputation**		
Yes		7 (26.9)
No		19 (73.1)

**Table 2:** Results of the evaluation of the ICG angiography data.

Variables	Mean ± SD (Min–Max)	n (%)
ICG Intensity Value	165.58 ± 27.6 (123–236)	26 (100.0)
ICG Full Intensity Time (s)	27.46 ± 6.4 (17–45)	26 (100.0)
Time to Wound Healing (days)	147.12 ± 107.36 (30–365)	24 (100.0)
Disease-free follow-up duration (days)	469.17 ± 206.48 (30–835)	24 (100.0)

**Table 3:** Correlations between ICG angiography and continuous variables.

	ICG Full Intensity Time (s)	Time to Wound Healing (Days)	Disease-Free Follow-Up Duration	ICG Intensity Value
**ICG Full Intensity Time (s)**	1			
**Time to wound healing (days)**	−0.177	1		
**Disease-free follow-up duration**	−0.186	−247	1	
**ICG intensity value**	0.159	−0.204	−0.23	1

## Data Availability

The data presented in this study are available on request from the corresponding author.

## References

[1] Wang K., Wang Y., Shi W., Shen K., Tao K., Ling R., Huang Y., Fu X., Hu D. (2024). Diagnosis and Treatment of Diabetic Foot Ulcer Complicated with Lower Extremity Vasculopathy: Consensus Recommendation from The Chinese Medical Association (Cma), Chinese Medical Doctor Association (Cmda). Diabetes Metab. Res. Rev..

[2] Schaper N.C., Van Netten J.J., Apelqvist J., Bus S.A., Fitridge R., Game F., Monteiro-Soares M., Senneville E., IWGDF Editorial Board (2024). Practical Guidelines On the Prevention and Management of Diabetes-Related Foot Disease (Iwgdf 2023 Update). Diabetes Metab. Res. Rev..

[3] Olin J.W. (2000). Thromboangiitis Obliterans (Buerger’s Disease). N. Engl. J. Med..

[4] Dargon P.T., Landry G.J. (2012). Buerger’s Disease. Ann. Vasc. Surg..

[5] Dimmick S.J., Goh A.C., Cauzza E., Steinbach L.S., Baumgartner I., Stauffer E., Voegelin E., Anderson S.E. (2012). Imaging Appearances of Buerger’s Disease Complications İn The Upper And Lower Limbs. Clin. Radiol..

[6] Fazeli B., Poredos P., Kozak M., Pecsvarady Z., Catalano M., Al Salman M.M., Altarazi L., Ali A.A., Bashar A.H., Bozkurt K. (2023). Diagnostic Criteria for Buerger’s Disease: International Consensus of Vas-European Independent Foundation İn Angiology/Vascular Medicine. Int. Angiol..

[7] Shionoya S. (1998). Diagnostic Criteria Of Buerger’s Disease. Int. J. Cardiol..

[8] Vijayakumar A., Tiwari R., Kumar Prabhuswamy V. (2013). Thromboangiitis Obliterans (Buerger’s Disease)-Current Practices. Int. J. Inflam..

[9] Mavioğlu L., Mungan U., Özeke Ö., Ertan Ç., Özatik M.A. (2013). Buerger’s Disease (Thromboangiitis Obliterans) with an Atypical Presentation: A Case Report. Turk. J. Thorac. Cardiovasc. Surg..

[10] Mills J.L., Taylor L.M., Porter J.M. (1987). Buerger’s disease in the modern era. Am. J. Surg..

[11] Piazza G., Creager M. (2010). Thromboangiitis Obliterans. Circulation.

[12] Ghirardini F., Martini R. (2024). Current Opinion on Diagnosis of Peripheral Artery Disease in Diabetic Patients. Medicina.

[13] Misra S., Shishehbor M.H., Takahashi E.A., Aronow H.D., Brewster L.P., Bunte M.C., Kim E.S.H., Lindner J.R., Rich K., American Heart Association Council on Peripheral Vascular Disease (2019). Perfusion Assessment in Critical Limb Ischemia: Principles for Understanding and the Development of Evidence and Evaluation of Devices: A Scientific Statement from the American Heart Association. Circulation.

[14] Conte M.S., Bradbury A.W., Kolh P., White J.V., Dick F., Fitridge R., Mills J.L., Ricco J.B., Suresh K.R., Murad M.H. (2019). Global Vascular Guidelines on The Management of Chronic Limb-Threatening İschemia. Eur. J. Vasc. Endovasc. Surg..

[15] Leenstra B., Wijnand J., Verhoeven B., Koning O., Teraa M., Verhaar M.C., De Borst G.J. (2020). Applicability of Transcutaneous Oxygen Tension Measurement in The Assessment of Chronic Limb-Threatening Ischemia. Angiology.

[16] Catella J., Long A., Mazzolai L. (2021). What Is Currently The Role of Tcpo2 in The Choice of The Amputation Level of Lower Limbs? A Comprehensive Review. J. Clin. Med..

[17] Met R., Bipat S., Legemate D.A., Reekers J.A., Koelemay M.J. (2009). Diagnostic performance of computed tomography angiography in peripheral arterial disease: A systematic review and meta-analysis. JAMA.

[18] Healy D.A., Boyle E.M., Clarke Moloney M., Hodnett P.A., Scanlon T., Grace P.A., Walsh S.R. (2013). Contrast-enhanced magnetic resonance angiography in diabetic patients with infra-genicular peripheral arterial disease: A systematic review. Int. J. Surg..

[19] Spittell J.A. (1983). Tromboangiitis Obliterans: An Autoimmune Disorder?. N. Engl. Med..

[20] Karakoyun R., Köksoy C., Şener Z., Gündüz U., Karakaş B., Karakoyun M. (2014). Comparison of Quality of Life in Patients with Peripheral Arterial Disease Caused by Atherosclerosis Obliterans or Buerger’s Disease. Cardiovasc. J. Afr..

[21] Van Den Hoven P., Ooms S., Van Manen L., Van Der Bogt Kea Van Schaik J., Hamming J.F., Vahrmeijer A.L., Van Der Vorst Jr Mieog J.S.D. (2019). A Systematic Review of The Use of Near-İnfrared Fluorescence İmaging in Patients with Peripheral Artery Disease. J. Vasc. Surg..

[22] Mothes H., Dönicke T., Friedel R., Simon M., Markgraf E., Bach O. (2004). Clinical use of indocyanine green fluorescence video angiography to assess tissue perfusion in microsurgery. J. Trauma Acute Care Surg..

[23] Choi B., Jang S.Y., Kim S.K., Kim N., Kim K., Kim D.K. (2020). The İncidence, Prevalence, And Survival Rate of Thromboangiitis Obliterans in Korea: A Retrospective Population-Based Study. Cardiovasc. Diagn. Ther..

[24] Fiessinger J.N., Frank M. (2015). Maladie de Buerger [Thromboangııtıs Oblıterans (Buerger’s Dısease)]. Rev. Prat..

[25] Chen Q., Chen J., Li J., Cheng Y., Zhang R., Liu Z. (2023). Recent Advances of Oxidative Stress in Thromboangiitis Obliterans: Biomolecular Mechanisms, Biomarkers, Sources and Clinical Applications. Thromb. Res..

[26] Kawarada O., Kume T., Ayabe S., Nakaya T., Nakai M., Nishimura K., Noguchi T., Yokoi Y., Ogawa H., Yasuda S. (2017). Endovascular Therapy Outcomes and Intravascular Ultrasound Findings in Thromboangiitis Obliterans (Buerger’s Disease). J. Endovasc. Ther..

[27] Firat A., Igus B. (2019). Endovascular Recanalization of Thromboangiitis Obliterans (Buerger’s Disease) in Twenty-Eight Consecutive Patients And Combined Antegrade-Retrograde Intervention in Eight Patients. Cardiovasc. Interv. Radiol..

[28] Wilke B.K., Schultz D.S., Huayllani M.T., Boczar D., Spaulding A.C., Sherman C.E., Murray P.M., Forte A.J. (2021). Intraoperative Indocyanine Green Fluorescence Angiography Is Sensitive for Predicting Postoperative Wound Complications in Soft-Tissue Sarcoma Surgery. J. Am. Acad. Orthop. Surg..

[29] Zimmermann A., Roenneberg C., Wendorff H., Holzbach T., Giunta R.E., Eckstein H.H. (2010). Early Postoperative Detection of Tissue Necrosis İn Amputation Stumps with İndocyanine Green Fluorescence Angiography. Vasc. Endovasc. Surg..

